# Synergistic Sealing of the Pleural Space: Combining Thoracoscopic Talc Poudrage and IPC for Malignant Pleural Effusion—A Case Series

**DOI:** 10.1002/rcr2.70331

**Published:** 2025-09-02

**Authors:** Mas Fazlin Mohamad Jailaini, Azat Azrai Azmel, Mohd Zulkifli Mohd Zain, Mohamed Faisal Abdul Hamid

**Affiliations:** ^1^ Respiratory Unit Hospital Canselor Tuanku Muhriz, Universiti Kebangsaan Malaysia Kuala Lumpur Malaysia; ^2^ Endoscopy Services Centre Hospital Canselor Tuanku Muhriz, Universiti Kebangsaan Malaysia Kuala Lumpur Malaysia

**Keywords:** indwelling pleural catheter, malignant pleural effusion, pleurodesis, pleuroscopy, talc poudrage

## Abstract

Management of malignant pleural effusion (MPE) via medical thoracoscopy presents a clinical challenge, particularly when deciding whether to proceed with talc poudrage during the same setting. This decision is often complicated by uncertainty about lung re‐expansion and the potential failure of pleurodesis. We describe a series of three patients with cancer‐associated MPE who underwent medical thoracoscopy with talc poudrage combined with indwelling pleural catheter (IPC) insertion. This combined approach served as a practical and effective strategy, offering immediate symptom relief, facilitating early pleural symphysis when feasible, and providing a reliable fallback option in cases of non‐expanding lung. The dual‐modality technique highlights a safe, flexible pathway for managing MPE with improved procedural confidence and patient‐centred outcomes. We report 3 cases of MPE successfully achieving pleurodesis with a combination of talc poudrage and IPC, resulting in removal of IPC on follow‐up.

## Graphical abstract

We describe the technique of performing talc poudrage and indwelling pleural catheter (IPC) insertion during the same setting to enhance procedural efficiency and patient outcomes. We report a case series of malignant pleural effusion demonstrating successful pleurodesis within 2–4 weeks using this combined approach.

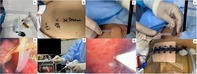

## 1 Introduction

Malignant pleural effusion (MPE) is a common and debilitating complication of advanced malignancy. It is associated with significant morbidity due to dyspnea and frequent fluid re‐accumulation, often requiring repeated interventions. Medical thoracoscopy has become a valuable tool for diagnostic assessment and therapeutic management of MPE. Talc pleurodesis remains a widely used method for achieving fluid control; however, its success is contingent on adequate lung re‐expansion. In cases where lung entrapment or non‐expansion is suspected, proceeding with talc poudrage during the same procedure poses a clinical dilemma, given the risk of pleurodesis failure. An indwelling pleural catheter (IPC), combined with talc poudrage, has emerged as a practical solution to this challenge. In this series, we describe three patients with MPE who underwent combined medical thoracoscopy, talc poudrage, and IPC insertion, offering definitive management and procedural flexibility.

## 2 Case Series

### 2.1 Case 1

**FIGURE 1:**
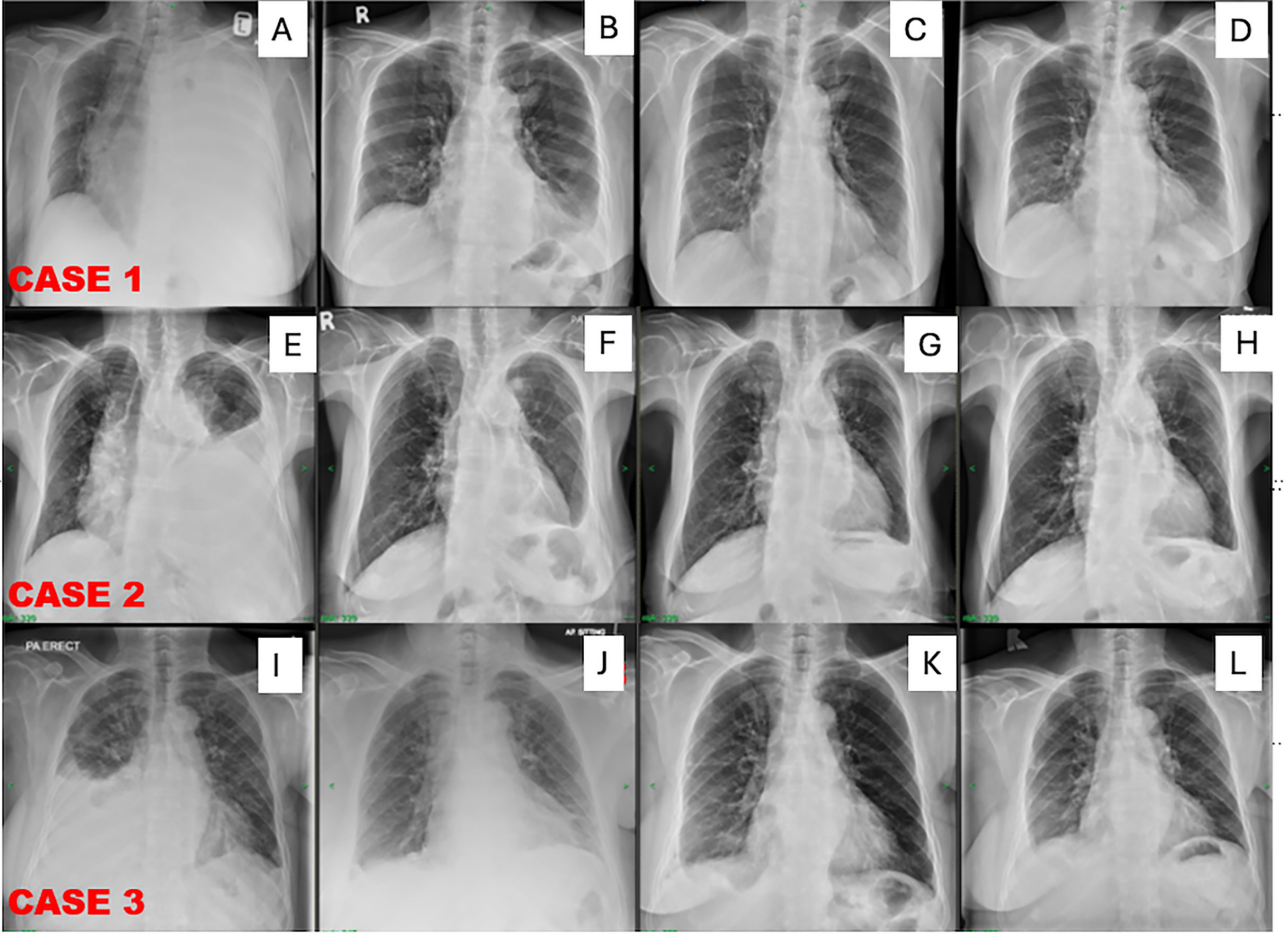
For case 1, CXR on initial presentation, showing left pleural effusion (A), attained spontaneous pleurodesis after 3 weeks and IPC removed (B), CXR after 1 month (C) and 3 month (D) after IPC removed showed no recurrence of pleural effusion. For case 2, CXR on initial presentation, showing left pleural effusion (E), attained spontaneous pleurodesis after 4 weeks and IPC removed (F), CXR after 1 month (G) 3 month (H) after IPC removed showed no recurrence of pleural effusion. For case 3, CXR on initial presentation, showing right pleural effusion (I), attained spontaneous pleurodesis after 2 weeks and IPC removed (J), CXR after 1 month (K) and 3 month (L) after IPC removed showed no recurrence of pleural effusion.

A 72‐year‐old woman with a history of hypertension and dyslipidemia, and a surgical history of total abdominal hysterectomy with bilateral salpingo‐oophorectomy (TAHBSO) for uterine fibroid, presented with progressive dyspnea and a non‐productive cough over 3 weeks, along with significant weight loss in the last 2 months. Initial chest X‐ray (CXR) showed complete left hemithorax opacification, indicating a massive left pleural effusion (Figure 1A). A 32 French chest tube was inserted, draining 1.8 L of fluid, which was analysed as an exudate with atypical cells suggestive of malignancy. Contrast‐enhanced computed tomography (CECT) imaging revealed a pleural‐based soft tissue mass in the left upper lobe and a large pleural effusion. A CT‐guided biopsy confirmed pulmonary adenocarcinoma, positive for TTF‐1 and CK7, with an EGFR exon 19 deletion. The patient began EGFR‐targeted therapy (Osimertinib). Six weeks later, pleural effusion recurred, leading to medical thoracoscopy for pleural biopsies and placement of an indwelling pleural catheter (IPC) with talc poudrage insufflation. During pleuroscopy, the lung appeared expandable, evidenced by full re‐expansion during suction and the absence of any significant adhesions or nodularity. Recovery was uneventful, and drainage decreased from 100 to 150 mL/day to 30 to 50 mL/day over 2 weeks. Patient attained pleurodesis after 3 weeks, and the IPC was removed (Figure 1B). There was no recurrence of the effusion noted during follow‐up at 1 month (Figure 1C) and 3 months (Figure 1D) after IPC removal.

### 2.2 Case 2

An 80‐year‐old woman with a medical history of hypertension and dyslipidemia presented with a 1‐month history of hoarseness of voice. An initial evaluation at a private hospital confirmed the diagnosis of advanced lung adenocarcinoma with malignant pleural effusion (MPE). She was subsequently referred to our oncology team, where targeted therapy with Gefitinib was initiated. For the management of her MPE, she was referred to our respiratory unit.

Upon assessment, her Eastern Cooperative Oncology Group (ECOG) performance status was 2, with a respiratory rate of 24 breaths per minute. Physical examination revealed markedly reduced breath sounds over the entire left hemithorax and stony dullness on percussion. CXR demonstrated a homogeneous opacity of the left lung with a contralateral mediastinal shift, consistent with a pleural effusion (Figure 1E). She underwent medical thoracoscopy, which included the insufflation of 4 g of sterile talc poudrage and the concurrent insertion of an indwelling pleural catheter (IPC) during the same procedure. During the procedure, visceral pleural nodules were noted, initially raising concern for a non‐expandable lung. However, the lung expanded well following radiological assessment the next day. The procedure was uneventful, and she was discharged 2 days later with appropriate IPC care instructions provided to her primary caregiver.

At home, the IPC was drained once daily, with pleural output progressively decreasing from the first week post‐procedure. By the second week, daily drainage was less than 50 mL, fulfilling the British Thoracic Society (BTS) criteria for successful pleurodesis. The IPC was removed within 1 month (Figure 1F). Follow‐up at 1 and 3 months, including serial chest radiographs and bedside thoracic ultrasound, confirmed no recurrence of pleural effusion (Figure 1G,H).

### 2.3 Case 3

A 67‐year‐old woman with a history of hypertension, dyslipidemia, and atrial fibrillation was diagnosed with left‐sided breast carcinoma, leading to a left mastectomy. Following her surgery, she initiated hormonal therapy and had several years of clinical stability. However, she subsequently experienced recurrent right‐sided pleural effusion (Figure 1I), necessitating multiple therapeutic thoracenteses. Pleural fluid cytology revealed malignant cells indicative of metastatic carcinoma; unfortunately, the cell count was too low for further immunohistochemistry (IHC) staining to identify the tissue origin. A subsequent Positron Emission Tomography (PET) scan uncovered hypermetabolic pleural‐based nodules, yet revealed no abnormal fluorodeoxyglucose (FDG) uptake in her breast or other areas, complicating the diagnostic process.

Faced with ongoing effusion and an unclear origin, we proceeded with a pleuroscopy. During this procedure, 4 g of sterile talc were used for talc poudrage, and an indwelling pleural catheter (IPC) was also inserted. Pleuroscopy demonstrated good lung reexpansion without any notable loculations or pleural abnormalities, supporting the assessment of an expandable lung. The pleural biopsy samples taken during this time confirmed the diagnosis of metastatic breast carcinoma. The procedure was successful, and she was discharged a few days later after her daughter received thorough education on IPC home care management. At home, the patient performed daily drainage of the pleural fluid. While the initial output was moderate, it progressively diminished over time. At her routine follow‐up visit 2 weeks post‐IPC insertion, significant drainage reduction was noted, leading to a clinical decision to remove the IPC after achieving successful pleurodesis at 3 weeks (Figure 1J). She remained asymptomatic upon follow‐up after IPC removal; and there were no clinical or radiological signs of pleural fluid re‐accumulation (Figure 1K,L).

## 3 Discussion

The management of MPE requires a multimodal approach tailored to the individual patient. Studies such as TIME2 and AMPLE demonstrate the advantages of indwelling pleural catheters (IPC) over talc pleurodesis, particularly in reducing hospital stay lengths and preventing further pleural interventions [1, 2]. Combining IPC with talc has shown benefits in achieving faster and more effective pleurodesis outcomes [3]. A recent meta‐analysis comparing pleurodesis techniques: talc slurry versus talc poudrage indicates that both methods are largely equivalent regarding pleurodesis success rates [4]. However, talc poudrage is associated with a higher incidence of respiratory complications, including pneumonia, empyema, pulmonary edema, and bronchopleural fistula formation [5]. Despite these risks, talc poudrage allows for direct visualisation of the pleural space during thoracoscopy, promoting uniform talc distribution on pleural surfaces. This visualisation may reduce the risk of loculated effusions and pleural adhesions, potentially improving long‐term patient outcomes.

Managing MPE in patients with a non‐expanding lung during medical thoracoscopy remains challenging, as the choice to perform talc poudrage is complicated by concerns regarding pleurodesis failure. The TACTIC trial has explored protocols suggesting that simultaneous IPC insertion during thoracoscopic talc poudrage is an area for ongoing research [6]. This aligns with international guidelines: the British Thoracic Society (BTS) recommends talc poudrage during thoracoscopy when biopsy is needed, reserving IPC for non‐expanding lung cases or failed pleurodesis [7]. Our case series contributes to this evidence by demonstrating that the combined approach of talc poudrage and IPC insertion can provide rapid symptom relief, offer management flexibility, and effectively control pleural effusion.

**FIGURE 2:**
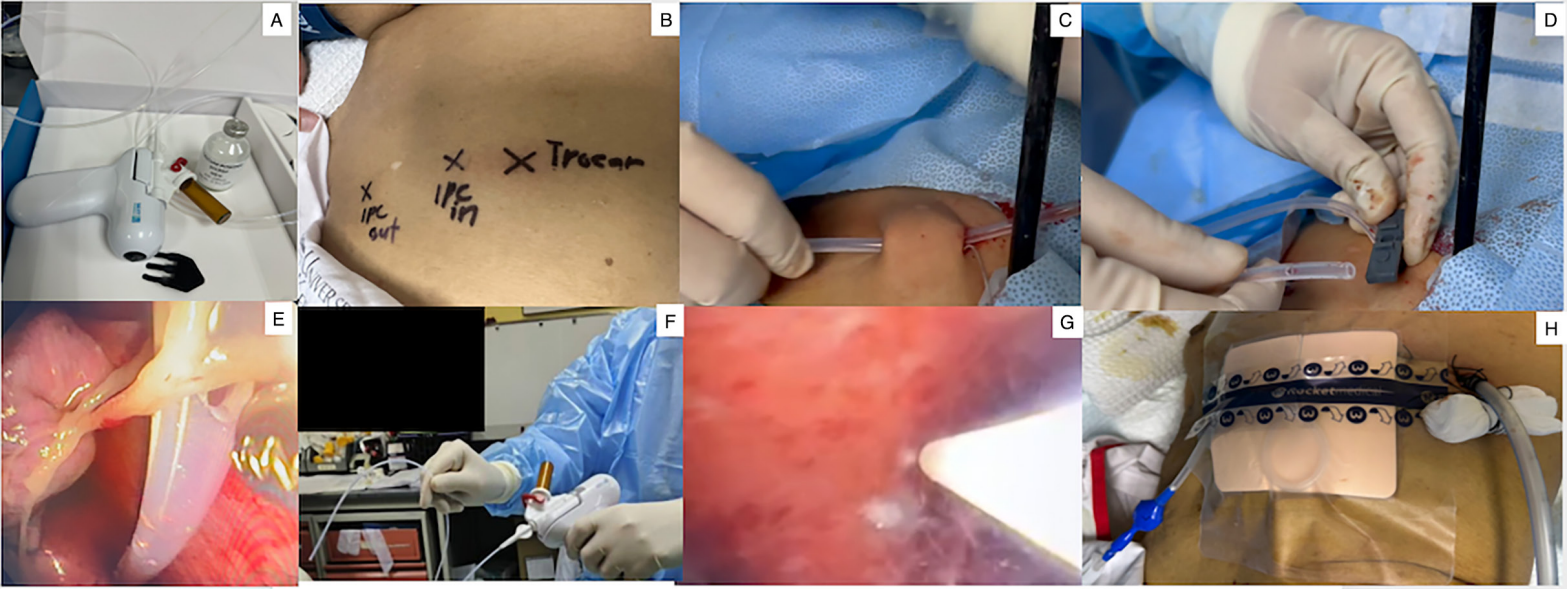
Air compressor device used for the talc poudrage (A). The pleuroscope entry point, IPC insertion site, and IPC exit site are marked on the patient's skin surface (B). A second operator concurrently inserts the IPC during pleuroscope (C and D). Fibrinous adhesions surrounding the IPC are cleared following completion of pleural biopsies and suctioning of pleural fluid (E). Talc poudrage catheter that will be inserted through the working channel of the pleuroscope (F). Talc insufflation into the pleural space (G). Two drainage tubes are in place: a 24 Fr chest tube and the IPC (H).

At our center, talc insufflation is performed using an air compressor device (Figure 2A). Prior to the procedure, the pleuroscope entry point, IPC insertion site, and IPC exit site are carefully marked on the patient's skin surface (Figure 2B). The procedure begins with pleural inspection using a pleuroscope. While one operator performs pleuroscopy and obtains pleural biopsies, a second operator concurrently inserts the IPC (Figure 2C,D).

Following completion of pleural biopsies and suctioning of pleural fluid, the position of the IPC within the pleural cavity is inspected, and any fibrinous adhesions surrounding the catheter are cleared (Figure 2E). Talc poudrage is then administered via a catheter inserted through the working channel of the pleuroscope (Figure 2F), which is connected to an air compressor. The talc canister is mounted onto the device, and compressed air propels the talc into the pleural space (Figure 2G). Throughout the procedure, the IPC remains connected to an underwater seal system to allow continuous evacuation of air. At the conclusion of the procedure, two drainage tubes are in place: a 24 Fr chest tube and the IPC (Figure 2H). Post‐procedure, the 24 Fr chest tube is maintained for 24 h and subsequently removed. Patients are typically discharged within 24–48 h, following instruction in IPC care and drainage technique.

A previous study supports our findings, with reported discharges within 1–3 days and pleurodesis success rates exceeding 75% [2]. A significant clinical consideration is whether talc slurry could serve as a viable alternative to talc poudrage following thoracoscopy. Importantly, this dual approach helps to balance the higher pleurodesis rates associated with talc poudrage and the fewer reinterventions seen with IPC‐only strategies, as supported by the TACTIC trial protocol. Our clinical experiences align with these observations, reinforcing the premise that thoracoscopic talc poudrage combined with concurrent IPC not only provides a flexible and patient‐centered option but also aligns with evolving pleural management guidelines.

This case series underscores the clinical value of integrating medical thoracoscopy, talc poudrage, and IPC insertion in the management of malignant pleural effusion. In cases where lung re‐expansion is uncertain, this dual approach offers both therapeutic flexibility and procedural safety, allowing pleurodesis to be attempted without compromising patient outcomes in case of failure. Our findings suggest that this strategy enhances symptom control, reduces procedural burden, and optimises long‐term pleural management for patients with advanced malignancies.

## Author Contributions

Mas Fazlin Mohamad Jailaini and Azat Azrai Azmel were involved in data collection from patient records and prepared the draft of the manuscript. Mohd Zulkifli Mohd Zain contributed to the technical aspects of the procedural description. Mohamed Faisal Abdul Hamid edited the manuscript and revised it critically for important intellectual content. All authors revised the manuscript and approved the final version of the manuscript.

## Consent

The authors declare that written informed consent was obtained for the publication of this manuscript and accompanying images using the form provided by the Journal.

## Conflicts of Interest

The authors declare no conflicts of interest.

## Data Availability

The data that support the findings of this study are available from the corresponding author upon reasonable request.

## References

[1] H. E. Davies , E. K. Mishra , B. C. Kahan , et al., “Effect of an Indwelling Pleural Catheter vs Chest Tube and Talc Pleurodesis for Relieving Dyspnea in Patients With Malignant Pleural Effusion: The TIME2 Randomized Controlled Trial,” Journal of the American Medical Association 307, no. 22 (2012): 2383–2389. 22610520 10.1001/jama.2012.5535

[2] R. Thomas , E. T. Fysh , N. A. Smith , et al., “Effect of an Indwelling Pleural Catheter vs Talc Pleurodesis on Hospitalization Days in Patients With Malignant Pleural Effusion: The AMPLE Randomized Clinical Trial,” JAMA 318, no. 19 (2017): 1903–1912. 29164255 10.1001/jama.2017.17426 PMC5820726

[3] R. Bhatnagar , E. K. Keenan , A. J. Morley , et al., “Outpatient Talc Administration by Indwelling Pleural Catheter for Malignant Effusion,” New England Journal of Medicine 378, no. 14 (2018): 1313–1322. 29617585 10.1056/NEJMoa1716883

[4] R. Bhatnagar , H. E. Piotrowska , M. Laskawiec‐Szkonter , et al., “Effect of Thoracoscopic Talc Poudrage vs Talc Slurry via Chest Tube on Pleurodesis Failure Rate Among Patients With Malignant Pleural Effusions: A Randomized Clinical Trial,” JAMA 323, no. 1 (2020): 60–69. 31804680 10.1001/jama.2019.19997 PMC6990658

[5] S. Mummadi , A. Kumbam , and P. Y. Hahn , “Malignant Pleural Effusions and the Role of Talc Poudrage and Talc Slurry: A Systematic Review and Meta‐Analysis,” F1000Research 3 (2015): 254. 10.12688/f1000research.5538.1 PMC4382843 25878773

[6] A. Dipper , A. Sundaralingam , E. Hedley , et al., “The Randomised Thoracoscopic Talc Poudrage+ Indwelling Pleural Catheters Versus Thoracoscopic Talc Poudrage Only in Malignant Pleural Effusion Trial (TACTIC): Study Protocol for a Randomised Controlled Trial,” BMJ Open Respiratory Research 10, no. 1 (2023): e001682. 10.1136/bmjresp-2023-001682 PMC10230980 37253535

[7] K. Sivabalah , H. Balata , C. Craig , et al., “The 2023 British Thoracic Society Guideline for Pleural Disease Update on Malignant Pleural Effusion,” Journal of Respiration 4, no. 4 (2024): 210–222.

